# Biological Monitoring: Evidence for Reductions in Occupational Exposure and Risk

**DOI:** 10.3389/ftox.2022.836567

**Published:** 2022-03-14

**Authors:** Jackie Morton, Craig Sams, Elizabeth Leese, Fiona Garner, Shahwaiz Iqbal, Kate Jones

**Affiliations:** Health and Safety Executive, Harpur Hill, Buxton, United Kingdom

**Keywords:** trends, lead, mercury, benzene, SPMA, hexamethylene diisocyanate, occupational exposure, biomonitoring

## Abstract

**Aims:** The aim of this publication is to explore occupational exposure trends from biological monitoring data collected over a period of more than 20 years. The data is stored within the HSE database, which holds more than 950,000 results from 120,000 workers in 8,000 companies. The data were collated for all biological monitoring results for lead, mercury, benzene, and hexamethylene diisocyanate exposures where there have been some regulatory drivers within the reported time period of the data searched.

**Methods:** Relevant results from sample analysed were extracted from the database and categorised by year from 1996 to the end of 2019 for individual blood lead results and individual urine results for mercury, benzene, and hexamethylene diisocyanate. Results were classed by broad occupational sector where possible. Data were reported graphically by analytical biomarker result (as 90th percentile (P90)) and number of samples per year as well as with overall summary statistics. To look at longer-term trends, results were also evaluated as P90 over 6-year periods.

**Results:** In the period 1996–2019, 37,474 blood lead, 11,723 urinary mercury, 9,188 urinary S-phenylmercapturic acid (SPMA, benzene metabolite) and 21,955 urinary hexamethylene diamine (HDA, metabolite of hexamethylene diisocyanate, HDI) samples were analysed and reported. Over the time period the blood lead concentrations saw the P90 reduce from 53 μg/dl 1996) to 24 μg/dl in 2019; the P90 urinary mercury levels reduced from 13.7 μmol/mol creatinine to 2.1 μmol/mol creatinine and the P90 urinary SPMA levels reduced from 133.7 μmol/mol creatinine to 1.7 μmol/mol creatinine. For HDI the P90 results reduced from 2 µmol HDA/mol creatinine in 1996–2000 to 0.7 in 2005–2010 but levels have since increased to 1.0 µmol HDA/mol creatinine (2016–2019).

**Conclusion:** There is strong evidence of reductions in exposure of GB workers to lead, benzene and mercury from the data presented here. These reductions may reflect the impact of national, regional and global regulatory action to reduce exposures however, the loss of high exposure industries (from either GB as a whole or just this dataset i.e., samples are being sent elsewhere) and the increase in automation or substitution also need to be considered as potential factors. The results for HDI show that whilst interventions can reduce exposures significantly, such initiatives may need to be refreshed at intervals to maintain the reductions in exposure. We have observed that exposures move between sectors over time. Waste and recycling (lead, mercury) and tunnelling through contaminated land (benzene) were sectors or tasks associated with significant exposures and may be increasingly areas of concern.

## Introduction

Globally there has been increasing concern and attention given to chemical exposures, particularly to the general population through environmental exposure. This has given rise to a number of initiatives and strategies to address these exposures and seek to reduce them over time. Some of these, such as the WHO International Programme on Chemical Safety, the United Nations Sustainable Development Goals (United Nations, 2021) and the Minamata Convention on mercury (Minamata Convention on Mercury, 2021) have been international efforts. The scopes of these initiatives range from raising awareness and harmonising approaches to, in the Minamata Convention, an outright ban on new mercury mines and commitments to reduce uses elsewhere.

The general principle that it is desirable to reduce chemical exposures also applies to occupational settings. Whilst many chemicals are assigned workplace exposure limits in national legislation around the world, there are also substances (particularly carcinogens, mutagens, sensitisers) where it is usually not possible to define safe levels, and therefore, concepts such as the precautionary principle and, in Great Britain, the use of ALARP (as low as reasonably practicable) dictate that reduced exposures are needed. Even in cases where workplace exposure limits exist, new toxicological data and new endpoints (such as the increased concern around endocrine disruption) can make revision and reduction of these limits advisable.

In terms of assessing trends in exposures both in environmental and occupational exposures it is useful to consider data collected over an extended time period. However, one of the problems of trying to look at trends in exposures is the lack of pooled data sources since most exposure data are held within the research or analysis institute or within the company that contracted the analysis. Although research data are usually published and thus available on certain platforms, they often represent cross-sectional surveys and there is unlikely to be harmonisation across different surveys conducted by different institutes. Routinely collected “compliance” data is unlikely to be published or pooled. Whilst there are initiatives to improve upon this situation [for example, IPChem (IPCheM Portal, 2021)], the goal of large, comparable exposure datasets is some way off.

When it comes to showing that a particular intervention has had an impact on exposure levels, this complicates the trend analysis further. It has been reported that exposures across many substances and industry sectors are decreasing on an annual basis (Creely et al., 2007) but contextual data to clarify the causes of the reductions is often absent and the lack of harmonisation of study designs, sampling and analysis adds uncertainty to any conclusions.

The Health and Safety Executive (HSE) operates the leading laboratory in Great Britain (GB) for biological monitoring, assessing chemical exposure *via* uptake and elimination of the substance (or a metabolite) in biological media (usually blood or urine). The focus is on occupational exposures although research involving general population exposures is also undertaken. Biological monitoring samples are collected from workers and are sent to the laboratory *via* HSE’s inspectors, hygienists and external occupational health professionals for investigations, research or routine monitoring. Since 1996 all results have been stored in a database that now has over 950,000 results from >120,000 workers in more than 8,000 companies based in GB (<2% of samples and companies are from overseas).

The aim of this publication is to explore occupational exposure trends from biological monitoring data stored within the HSE database. Therefore the database was searched for occupational biological monitoring results for lead, mercury, benzene, and hexamethylene diisocyanate exposures where there have been some regulatory drivers within the reported time period of the data searched. Blood lead measurements have been required under the Control of Lead at Work Regulations since 1980 where workers have the potential for ‘significant exposure’; with the latest revision dating from 2002 (Health and Safety Executive, 2002); action levels and suspension levels are in force to limit exposures although these have not been revised since 1998. Mercury has been the subject of global pressure to reduce uses and exposures, culminating in the agreement of the Minamata Convention in 2013, which came into force in 2017. HSE set a health-based biological monitoring guidance value (BMGV) for mercury in urine in 1995 (Health and Safety Executive, 1996). Benzene has long been recognised as a carcinogen and occupational exposure limits around the world have declined steadily (for instance, the ACGIH TLV has reduced from 100 ppm (8 h TWA) in 1946 to 0.5 ppm in 1997, retained in 2001 (ACGIH., 2020)). Diisocyanates have been a leading cause of occupational asthma in GB for many years. At the turn of the 21st century, motor vehicle spray painters were identified as 80 times more likely than any other occupation to have occupational asthma. In response, HSE implemented a national intervention of Safety and Health Awareness Days with over 4,000 attendees at 32 events (Piney et al., 2015). This was shown to have reduced exposures to HDI (the most common diisocyanate used in spray paints) (Jones et al., 2013) and, consequently, HSE has recommended at least annual biological monitoring for paint sprayers (HSE, 2012) and introduced a BMGV for all isocyanates in 2005.

The results for each substance were expressed as the 90th percentile values (P90) for each year. The purpose was to see if there were trends in the exposures for these particular compounds and whether there was any evidence for an impact of interventions. Whilst this data suffers from a lack of contextual information, it does represent longitudinal data from a large number of workers with sample analysis procedures applied consistently over time and with appropriate external quality assurance.

## Methods

### Sample Collection

The majority of samples are received as part of routine exposure monitoring, either directly from the company or *via* an occupational health provider. Most samples will be provided at the end of a typical shift although other time points are possible, depending on the sampling advice. It is the responsibility of the person managing the company’s biological monitoring programme to obtain and store informed consent from participating workers as per HSE’s guidance (HSE, 1997). Our sample request paperwork reminds them of this obligation and asks them to confirm but the database itself does not store evidence of consent.

### Biological Monitoring Database

The HSE biological monitoring database has been briefly described previously (Cocker and Jones, 2017). It is a Microsoft Access database that stores individual biomarker results per sample per worker per company (where available). It has been in use since 1996 and houses over 950,000 results from more than 120,000 workers in over 8,000 companies. The majority of samples are received as part of routine exposure monitoring, either directly from the company or *via* an occupational health provider; generally little or no contextual data is provided or stored in the database. Many samples are received with no information as to the type of company, industrial sector or form of exposure; for example when received from academic institutions, local government councils, occupational health providers and GPs and hospitals. The database also stores research results where there may be more information on tasks and control measures. In some cases this is stored within the database as questionnaire data but often contextual data is too narrative to be efficiently captured in a database system, particularly one designed to capture analytical results rather than occupational hygiene observations.

Queries were run within the database to extract results from samples analysed by year from 1996 to the end of 2019 for each selected substance. Companies were classed by sector where possible (it was not possible to retrospectively assign Standard Industrial Classification codes). Data was reported by analytical biomarker result (as P90) and number of samples per year.

### Biological Sample Analysis

Lead in blood and mercury in urine are determined by direct measurement of the analyte. The biological monitoring of benzene and hexamethylene diisocyanate in urine samples are determined by measuring specific biomarker metabolites, S-phenyl-mercapturic acid (SPMA) and 1,6 hexamethylene diamine (HDA) respectively. An overview of the analytical methodology is described below.

### Blood Lead Analysis

Since 2003 to date the analytical method for blood lead analysis has been using inductively coupled plasma mass spectrometry (ICP-MS) (Morton et al., 2010). The limit of quantification for this current method is 0.1 μg/dl (note the units for blood lead are µg/dL i.e., µg/100 ml). From 1996 to 2003, blood lead was determined using graphite-furnace atomic absorption spectrometry (GF-AAS) where the GF-AAS limit of quantification was 2.5 μg/dl. Using GF-AAS any low results were reported as <5 μg/dl. The certified reference materials (CRMs) analysed in each analysis are Bio-Rad Lyphocheck Levels 1 and 2 (Bio-Rad Laboratories Ltd., Watford, UK) and a typical inter-assay coefficient of variation is 6% For blood lead determination the laboratory also participates in external quality assessment schemes (EQA), currently UK NEQAS for Trace Elements (Guildford, UK). The analytical method is ISO: 17025 accredited.

### Urinary Mercury Analysis

The analysis of mercury in urine was undertaken using ICP-MS for the whole time period (Morton et al., 2014). CRMs used in each analysis are comprised of ClinChek Urine Control for Trace Elements levels 1 and 2 (Recipe, Germany) and historically Lypocheck Urine Metals Control levels 1 and 2 (Bio-Rad Laboratories, Watford, UK). The limit of quantification (LOQ) is 0.094 μg/L in undiluted urine and the inter-assay coefficient of variation for the method is 13%. The laboratory participates in external quality assurance schemes G-EQUAS (University Erlangen-Nuremberg, Germany) and previously UK-NEQAS for Trace Elements (Guildford, UK) and the method is ISO:17025 accredited. The urinary mercury UK biological monitoring guidance value (BMGV) is 20 μmol/mol of creatinine and the background reference range for a non-occupationally exposed population in the UK is <1.5 μmol/mol of creatinine (Morton et al., 2014).

### Urinary SPMA (for Benzene) Analysis

Exposure to benzene was assessed by quantifying the level of S-phenyl-mercapturic acid (SPMA) in urine. SPMA is a minor, but very specific phase II metabolite of benzene; arising from glutathione conjugation of the intermediate oxidative metabolite benzene oxide (Morton et al., 2014). Consequently, there is greater potential for inter-individual variability in SPMA levels in comparison to some of the other biomarkers reported here. Analysis is undertaken using liquid chromatography-tandem mass-spectrometry (LC-MS) after the samples are acidified and extracted (Jones and McCallum, 2011; Arnold et al., 2013).

Frozen aliquots of bulk spiked in-house QC samples were run with each batch of samples. The method has a limit of quantitation of 5 nmol/L (this has reduced over time with improved instrumentation) and an inter-assay coefficient of variation of 15%. The laboratory participates in an external quality assurance scheme (G-EQUAS, University Erlangen-Nuremberg, Germany).

### Urinary HDA (for Hexamethylene Diisocyanate) Analysis

The analytical method used for the determination of hexamethylene diisocyanate (HDI) in urine is based on GC-MS determination of urinary free dianiline metabolites after hydrolysis (Cocker et al., 2017). For these samples 1,6 hexamethylene diamine (HDA), a breakdown product of HDI, was analysed. The limit of quantitation is 5 nmol/L and the inter-assay coefficient of variation for HDA is 5.6% (Cocker et al., 2017). The laboratory participates in an external quality assurance scheme (G-EQUAS, University Erlangen-Nuremberg, Germany) and the method is ISO:17025 accredited. The BMGV in Great Britain is 1 μmol/mol creatinine.

### Urinary Creatinine

The three urine assays discussed here are all corrected for creatinine concentration before reporting; the samples are spot samples, usually taken at the end of shift and so hydration status needs to be considered. Creatinine was determined by an automated alkaline picrate method (Cocker et al., 2011), using an automated spectrophotometer (samples since 2005 were analysed on a Pentra 400 or C400, HORIBA ABX UK, Northampton, UK). An internal QC material made from a pooled urine sample and stored frozen in 1 ml aliquots was used, the inter-assay coefficient of variation is 1.9%. The detection limit of the method is 0.2 mmol/L; samples with creatinine values outside of the generally accepted “normal range” [3–30 nmol/L (World Health Organisation, 1996)] are flagged on the reports generated by the database but these data have not been excluded from this dataset. The laboratory participates at least monthly in an external QA scheme (RIQAS)^1^ and the method is ISO:17025 accredited.

## Results

**FIGURE 1:**
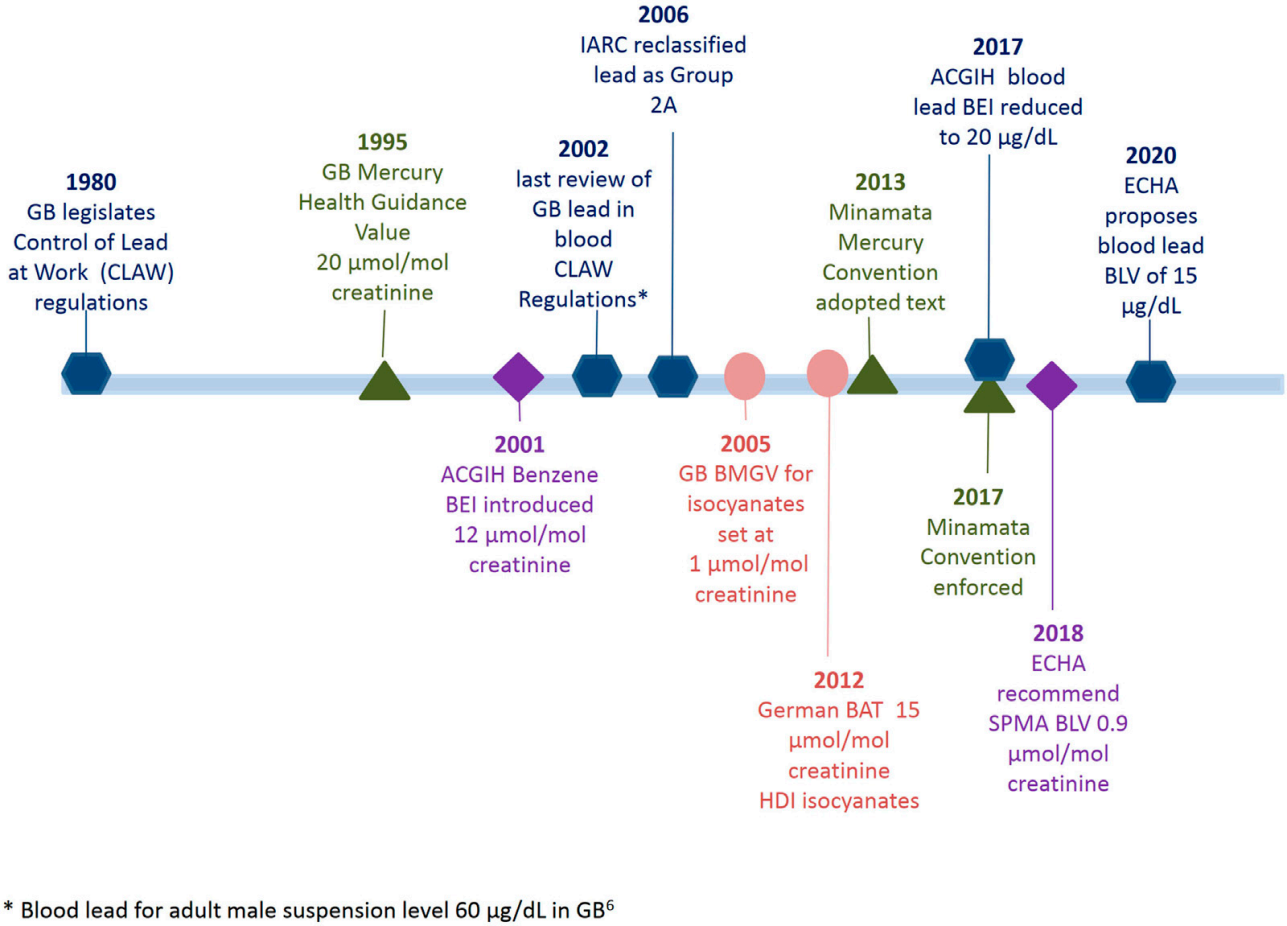
Timeline of regulatory acts and biological monitoring guidance value updates for blood lead, urinary mercury, urinary benzene (as SPMA metabolite) and urinary isocyanates (as HDA).

Summary statistics covering the time period 1996–2019 in 6 year intervals for each substance can be found in Table 1. To assist with comparing the guidance levels and regulatory interventions in Europe, the United States of America and Great Britain a timeline is outlined in Figure 1.

**TABLE 1:** Summary statistics in four time periods for blood lead and urinary mercury, benzene (as SPMA) and hexamethylene diisocyanate data.

Lead	1996-2001	2002-2007	2008-2013	2014-2019
P50	24	17	12	6
P75	41	33	26	16
P90	53	45	38	29
P95	59	51	44	36
Number of samples	9559	9641	11031	7183

Mercury	1996-2001	2002-2007	2008-2013	2014-2019
P50	1.6	0.9	0.6	0.5
P75	8.7	2.4	1.6	1.1
P90	17.6	6.2	4.8	2.5
P95	24.4	10.6	8.5	4.0
Number of samples	2305	1993	4732	2694

SPMA	1997*-2001	2002-2007	2008-2013	2014-2019
P50	3.1	1.7	<LOQ	<LOQ
P75	8.1	4.0	1.8	0.7
P90	26.9	8.4	4.3	2.0
P95	91.6	12.9	7.9	2.9
Number of samples	872	898	4613	2805

Hexamethylene diisocyanate	1996-2001	2002-2007	2008-2013	2014-2019
P50	<LOQ	<LOQ	<LOQ	<LOQ
P75	0.7	<LOQ	<LOQ	<LOQ
P90	2.2	0.7	0.7	1.0
P95	5.0	1.4	1.2	1.9
Number of samples	206	2868	9484	9397

*Note the five year time period, SPMA analysis began in 1997.

### Lead

Since 1996, the laboratory analysed between 900 and 2,100 blood lead samples per year, a total of 37,414 individual samples. Despite the sample numbers increasing slightly from 2018 to 2019, the numbers have generally reduced across the dataset since a peak in 2007, an overall reduction of 9% in the whole period of 1996–2019. In this 24 year period samples were received from industries including battery, smelting, casting and alloy manufacture and foundries; chemical and pigment and glass manufacturers; construction and demolition companies as well as roofing and glazing companies; scrap, recycling and decommissioning companies as well as from occupational health providers. It should be noted that sample numbers can in part be influenced by the increased frequency of sampling when a blood lead result is over 30 μg/dl (Health and Safety Executive, 2002).

**FIGURE 2:**
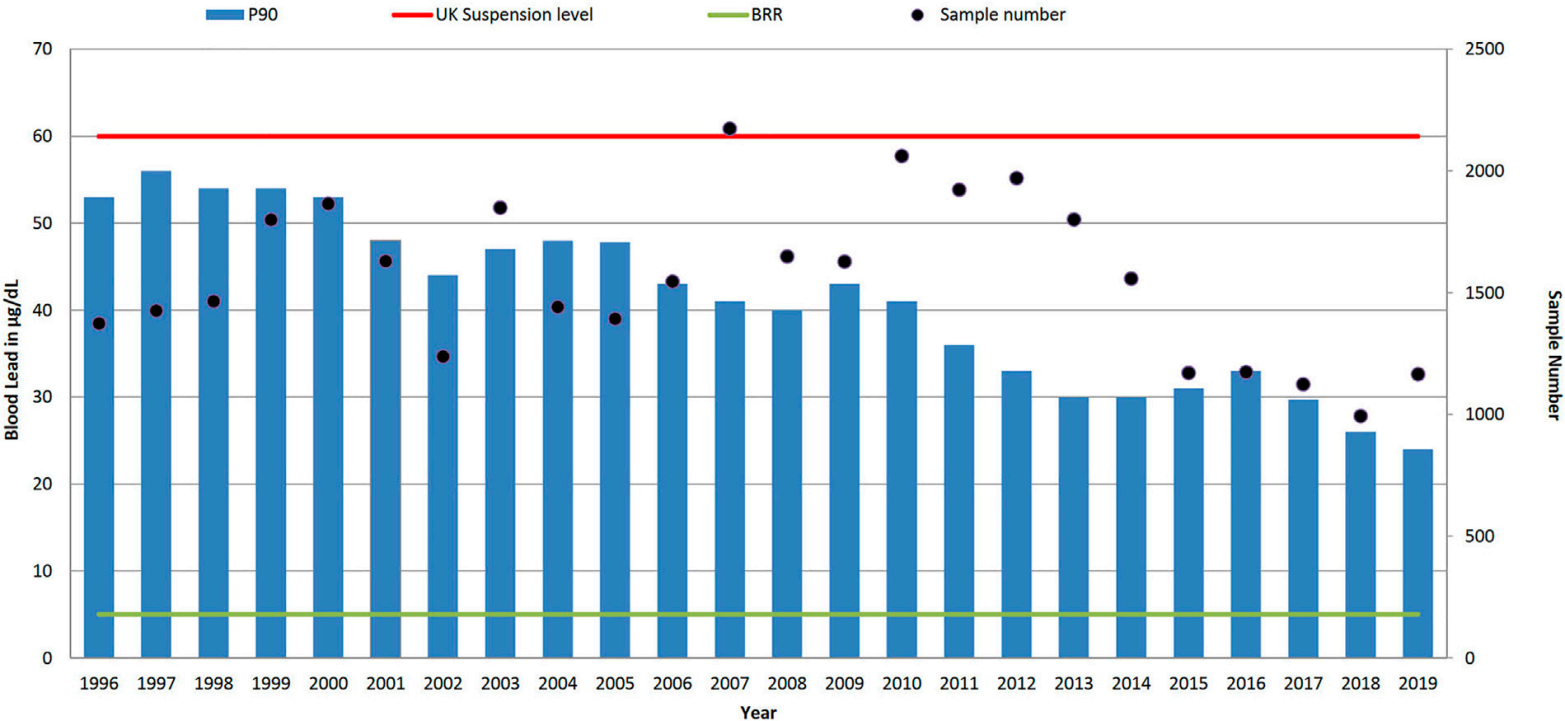
Trends in P90 blood lead level concentration (µg/dl) and sample numbers (• right-hand axis), 1996–2019. UK suspension limit of 60 μg/dl for adult males, and non-occupationally exposed background reference range (BRR) of 5 μg/dl.

As shown in Figure 2 the P90 value of blood lead concentration has steadily decreased from 53 μg/dl in 1996 to 24 μg/dl in 2019, with an overall reduction of 56%. The median (P50) values for blood lead have reduced tenfold from 27 μg/dl in 1995 to 3 μg/dl in 2019, which have followed the 3.1% year-on-year reduction trend as identified from this dataset in 2007^14^. Comparing the data across 6 years intervals, as shown in the summary statistics in Table 1, the P95 of the data decreases from 59 (1996–2001), to 51 (2002–2007), to 44 (2008–2013) to 36 μg/dl (2014–2019). However, it is evident from the recent data, where the latest 6 years P50 is 6 μg/dl, that whilst the number of samples with low lead concentrations dominates the summary data there still remains an exposed population of workers. In the most recent reported year, 2019, the industries with the highest blood lead P90s were smelting and casting (41 μg/dl 5.4% of all samples received); smelting and alloys (33 μg/dl 2.1% all samples); chemical and pigment manufacture (30 μg/dl 7.9% all samples); scrap and recycling (28 μg/dl 3.8% all samples); roofing and glazing (26 μg/dl 7.9% all samples); with construction and demolition exhibiting a P90 of between 10 μg/dl and 11 μg/dl respectively for 8% of the all samples in 2019. Almost half of the samples were received with little or no contextual data from occupational health providers (P90 16 μg/dl 24% all samples) and the ‘other’ category (P90 12 μg/dl 25% all samples).

### Mercury

Between January 1996 and December 2019, 11,724 urine samples for mercury analysis were received at the laboratory from approximately 289 different companies or sources. Samples were received from industries such as the waste and recycling sector, oil, gas and petroleum companies, plastic and rubber producers, production/maintenance of mercury-based measurement equipment (thermometers, barometers and sphygmomanometers), chemical production companies, metal production companies including copper alloy producers, electronics manufacturing and lighting maintenance and manufacturers including both light bulbs and neon sign producers. In addition, urine samples were also sent from crematoria, dental practices and companies involved with contaminated land and bioremediation.

The majority of urine samples over the 24 year period were received from the oil and gas industry accounting for 40% of all samples received (n = 4,634). The second largest source of samples was from companies who manufactured and/or maintained mercury based measurement equipment totalling 13% of all urine samples (n = 1,486). These 1,486 samples were sent by only nine different companies (3% of the total companies sending samples). No contextual information was sent with 25% of the urine samples received at the laboratory in the 24 year period (from 132 sources from a range of hospitals/doctors, occupational health companies, academia and other unknown sources).

**FIGURE 3:**
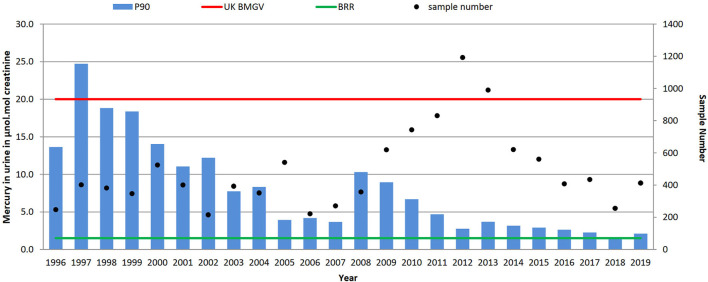
Trends in P90 urinary mercury concentrations (µmol/mol creatinine) and sample numbers (• right-hand axis), 1996–2019. UK biological monitoring guidance value 20 μmol/mol creatinine and non-occupationally exposed background reference range (BRR) of 1.4 μmol/mol creatinine.

As seen in Figure 3, a reduction of the P90 has been observed from a maximum of 24.7 μmol/mol creatinine (in 1997) to 2.1 μmol/mol creatinine in 2019. Only samples received in 1997 had a P90 above the UK biological guidance value of 20 μmol/mol creatinine, and as shown in Table 1 the P90 has declined from 17.6 (1996–2001), to 6.2 (2002–2007), to 4.8 (2008–2013) to 2.5 μmol/mol creatinine (2014–2019). However, there was at least one urinary mercury result above the UK guidance value annually for 17 of the 24 years, the highest being 203.8 μmol/mol creatinine in 2008 from a waste and recycling source. Since the 6 year period 2002–2007 the median urinary mercury level has been below the background reference range of 1.4 μmol/mol, reducing over the time period from 1.7 μmol/mol creatinine in 1996 to 0.4 μmol/mol creatinine in 2019; the highest median level (6.0 μmol/mol creatinine) was observed in 1997.

There is an interesting increase in P90 values between 2008 and 2010/2011 where it was noted that two companies sending samples for urinary mercury determinations influenced the increase of P90 observed. The samples were received from a metal recycling company and a petroleum based company neither of whom had previously submitted samples. The increase of P90 between 2007 and 2008 (3.7–10.3 μmol/mol creatinine) was as a result of the samples from the metal recycling company. They sent only 45 samples in 2008 but the P90 of these samples was 115.3 μmol/mol creatinine (min-max 1.8–203.8, median 36.6 μmol/mol creatinine). Then a sustained increase in P90 from 2009 was influenced by one petroleum company, with a P90 of 21.6 μmol/mol creatinine (n = 89 samples) evidenced in 2009. This level was then seen to incrementally reduce year on year within the company to a P90 of 9.2 μmol/mol creatinine (n = 153 samples) by 2011 and continued until 2019 when a P90 of 3.1 μmol/mol creatinine (n = 61 samples) was observed.

One industry that has consistently provided samples over the time period is the mercury containing instrument manufacturing industry. The mercury in urine results across the time period show that the industry did have significant exposure 1996–2000. However by 2016 workers exhibited mercury levels within the background reference range whilst sample numbers had reduced by 90%. This trend suggests that the industry has changed, reducing worker exposure (by improved control measures, substitution and/or more automation).

### Benzene

**FIGURE 4:**
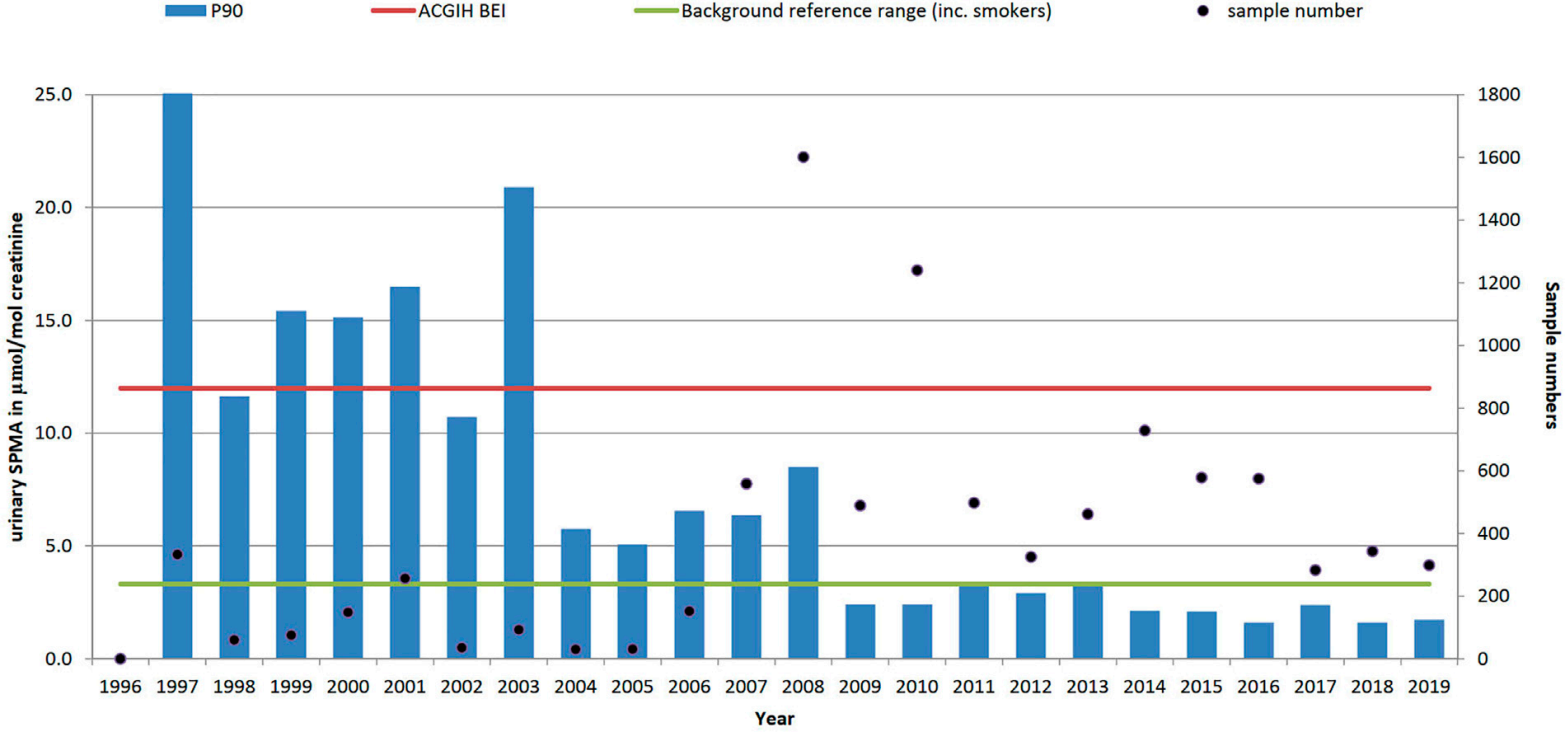
Trends in P90 urinary benzene metabolite level (SPMA, µmol/mol creatinine) and sample numbers (• right-hand axis), 1996–2019 (method not available in laboratory until 1997). ACGIH biological exposure index value 12 μmol/mol creatinine and non-occupationally exposed background reference range (BRR) of 3.3 μmol/mol creatinine.

Since 1997 (when the SPMA method was established at our laboratory) over 9,000 individual urine samples have been analysed for SPMA. Most samples were received from the petroleum industry (36%), coke oven workers (18%), foundries (6%) and waste and recycling (4%). The total number of samples received in any given year ranged from 30 in 2004 up to 1,601 in 2008. Sample numbers in 2008 were substantially boosted as a result of a contaminated land incident (Jones and McCallum, 2011) (a similar incident was also monitored in 1997, accounting for the high P90 in that year), but the median sample number of 461 since 2011 reflects the current levels of samples received from multiple sources on a commercial basis. Figure 4 shows that urinary SPMA levels have generally been decreasing over time, although more recent data suggests that levels may be stabilising. As shown in Table 1 when viewed over 6-years time periods, the P90 urinary SPMA levels show clear reductions from 26.9 μmol/mol creatinine (1997–2001); -8.4 (2002–2007); 4.3 (2008–2013); 2.0 (2014–2019. The large reduction in P90 from 2008 appears to be due in large part to the petroleum sector; samples numbers from this sector have increased markedly in recent years and yearly P90 values have been low (generally less than 2 μmol/mol creatinine).

These urinary SPMA data reflect total benzene exposure from all sources and, while self-reported smoking status has been collected, we have not attempted to classify the results according to smoking status. In the US, ACGIH have had a Biological Exposure Index for S-PMA as a marker of benzene exposure since 2001, reflecting the US TLV of 0.5 ppm (the current UK WEL is 1 ppm); in the absence of a UK BMGV, the BEI has been used as a benchmark in Figure 3.

### Hexamethylene Diisocyanate

**FIGURE 5:**
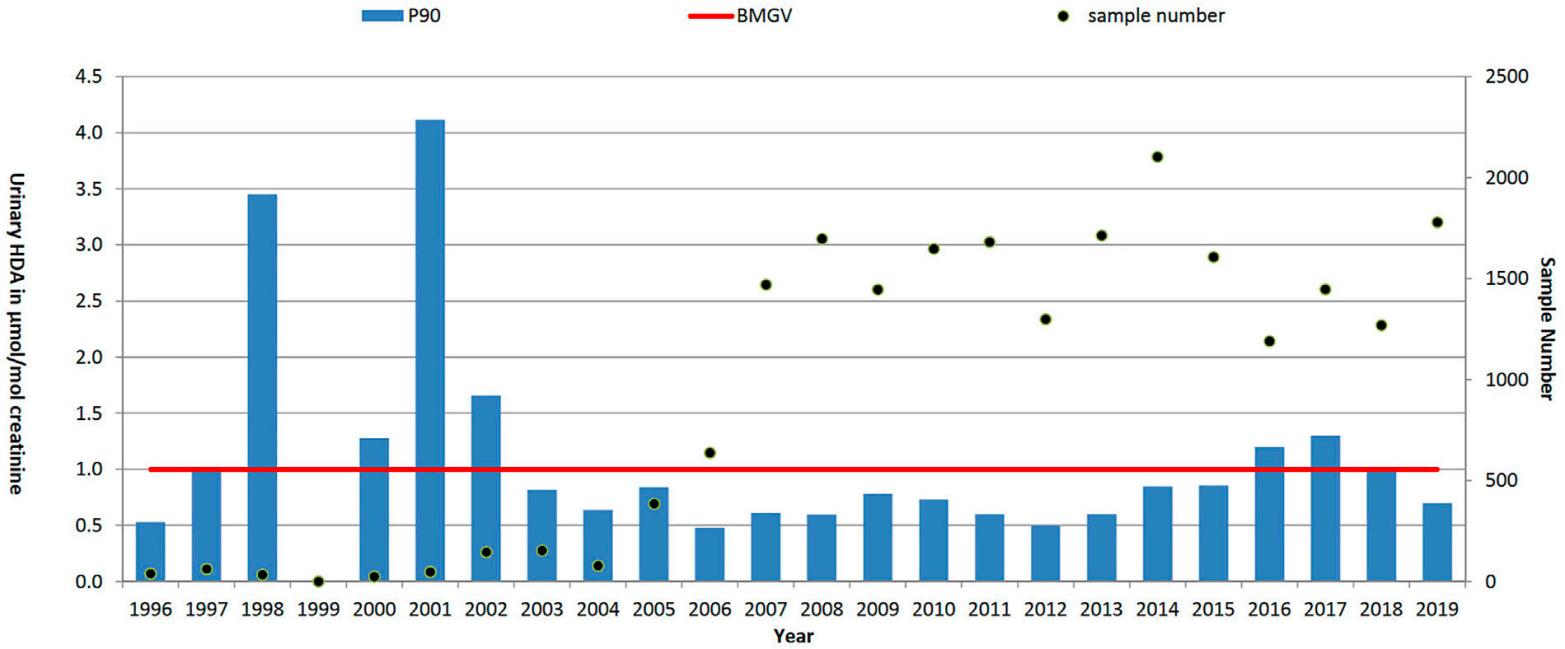
Trends in P90 urinary HDI metabolite level (HDA, µmol/mol creatinine) and sample numbers (• right-hand axis), 1996–2019. UK biological monitoring guidance value (BMGV) 1 μmol/mol creatinine and non-occupationally exposed persons have levels below the LOQ of the method. GB national intervention ran from 2004 to 2008.

For hexamethylene diisocyanate (HDI), with a total of 21,955 results reported here, were restricted to automotive manufacture and repair sectors, P90 results reduced (Table 1) from 2.2 µmol HDA/mol creatinine in 1996–2001 (prior to the national intervention study (Piney et al., 2015)) to 0.7 in 2008–2013 but levels have since increased to 1.0 µmol HDA/mol creatinine (2014–2019). The early P90 (1996–2001) may have been biased due to small numbers (∼35 samples per year) and a focus on regulatory investigations by HSE inspectors. Numbers increased substantially (as shown in Figure 5) during the intervention and have since remained at about 1,500 samples per year in these sectors.

## Discussion

We have presented general exposure trends for four substances where there has been some form of regulatory input over time, either a specific time-limited intervention (e.g., HDI) or ongoing legislation (see the timeline in Figure 1). The data have not been specifically acquired but rather reflect the samples received and analysed for biological monitoring purposes at the laboratory since 1996. As discussed previously (Cocker and Jones, 2017), it is not possible to say whether the sampling is biased, either to the better performers in the relevant sectors or to those with more severe exposure control issues. Nevertheless, taking the results at face value, it can be seen that generally exposures have declined over time; considering all data across 5-year time periods, blood lead levels decreased from 54 to 29 μg/dl, urinary mercury levels decreased from 18.6 to 2 μmol/mol creatinine, urinary SPMA levels decreased from 26.9 to 1.8 μmol/mol creatinine and urinary HDI levels decreased from 2.0 to 1.0 μmol/mol creatinine. These general trends agree with the observations of others (Creely et al., 2007); there are some exceptions such as mercury in the waste and recycling sector and this may highlight where future exposure problems might occur. In general sample numbers for lead, mercury and benzene have been sustained over time even though worker numbers within specific sectors may have declined or increased. HDI sample numbers have increased significantly due to the widespread use (there are an estimated 12,000 motor vehicle paint sprayers in the UK (Broughton et al., 2010)), and the strong recommendation from HSE for, at least annual, monitoring (HSE, 2012). Although lead is becoming less widespread as an exposure issue, the combination of the regulatory requirement for testing (Health and Safety Executive, 2002) and a reduction in available laboratories has meant that sample numbers at our laboratory have remained fairly steady.

### Lead

In Great Britain, the Control of Lead at Work (CLAW) Regulations stipulate that all workers with *significant* lead exposure, as defined, are required to undergo medical surveillance which includes measurement of blood-lead concentrations (Health and Safety Executive, 2002). The current UK suspension limits, unchanged since 1998, are 60 μg/dl for adult males, 50 μg/dl for younger workers (age 16–17 years) and 30 μg/dl for women of reproductive capacity. Whilst the action and suspension levels in both the GB CLAW (Health and Safety Executive, 2002) regulations and EU legislation (The Council of the European Union, 2021) (current Biological Limit Value (BLV) is 70 μg/dl and still used as such in six EU member countries) have not reduced, both the EU Scientific Committee on Occupational Exposure Limits (SCOEL) (SCOEL, 2021) and the American Conference of Governmental Industrial Hygienists (ACGIH) committee lowered their recommended action values for lead in blood to 30 μg/dl in 1995 and 2002, respectively. In 2017 the ACGIH committee reduced the Biological Exposure Index (BEI) to 20 μg/dl (SCOEL, 2021) and in 2020 the German Commission also adopted 20 μg/dL as the biological guidance at the workplace (BLW) level (Bolt et al., 2020). In 2017 the International Lead Association and its members agreed to set a voluntary target value where all employees would have a blood lead level lower than 20 μg/dl by 2020 (ILA News, 2017). The proposed reductions went further still when in October 2019 the European Chemicals Agency (ECHA) suggested a BLV of 150 μg Pb/L (15 μg/dl) in blood for both inorganic and organic lead compounds (Echa Scientific Report, 2021).

Within the reported dataset, in 2019 only one blood lead sample was above the UK suspension limit of 60 μg/dl compared to 5% (n = 63) of samples in 1996. In the most recent blood lead data in 2019 presented in Figure 2, 6% of results are above 30 μg/dl and 18% of blood lead results are currently above the ECHA proposed BLV of 15 μg/dl. If the observed year-on-year reduction of 3.1% in blood lead levels continued, it will be 2033 before the P90 of the samples analysed falls below 15 μg/dl. A similar trend for the reduction of lead exposures in lead workers has been seen in the US where the prevalence of blood lead results greater than 25 μg/dl declined from 14.0 per 100,000 employed adults in 1994 to 5.2 per 100,000 employed adults in 2013 (Alarcon, 2016).

The reason for the reduction of the blood lead limits are as a result of the increased evidence outlining the health risks involved at lower levels of blood lead values. Environmental lead concentrations have reduced considerably since the mid-1990s since the phased removal of lead in petrol was introduced as well as a combination of regulatory/policy changes (Tsai and Hatfield, 2011), public health campaigns and an overall decline in the commercial usage of lead (Tong et al., 2000). This has resulted in a reduction of lead exposures in the general populations in the developed world. This trend is demonstrated well within the US NHANES dataset, collated by the Centre of disease Control (CDC), where the median blood lead level in the general population (age >20 years) has reduced from 1.7 μg/dl in 1999/2000 to 0.88 μg/dl in 2015/2016 (Centres for Disease Control and Prevention, 2021). Recently, the German Commission for the Investigation of Health Hazards of Chemical Compounds in the Work Area has re-evaluated the biological reference value (BAR) for blood lead. The BAR (95th percentile) of 7 μg/dl for the adult general population was set in 2012 and this has now been reduced to 3 μg/dl for women and 4 μg/dl for men (data from 4,936 samples in the time period 2010–2019 (Göen et al., 2020)).

In the dataset presented here the most recent (2019) median blood lead level in samples analysed from 1,165 lead workers was 3 μg/dl, a significant decline from 31 μg/dl in 1995 and is within German BAR normal population range, of less than 4 μg/dl for men. Such low levels in workers would suggest that there has been a substantial reduction in workplace exposures and that there is a cohort of workers that are perhaps unnecessarily being placed under health surveillance if their levels are within the reference ranges.

The number of lead workers in the UK as a whole has also considerably reduced over the past 25 years. HSE’s annual data collected under the CLAW regulations shows an overall decrease of almost two thirds from 17975 lead workers in 1998/1999 to 5,875 lead workers in 2018/2019 (HSE, 2019). HSE data is compiled from HSE appointed doctors’ reports for lead workers under medical surveillance. Whilst the data from our laboratory certainly contributes to the HSE CLAW reports there are more than 20 other laboratories involved. However, overall the HSE CLAW data does often reflect similar trends to our dataset, for instance interestingly and against the recent observed trends, in 2018/19 HSE reported a 15% increase in worker numbers and our laboratory saw a 17% increase in sample numbers.

The fact that between 1995–2019 there have been both significant reductions in blood lead levels and the number of workers does not mean that occupational lead exposure can be dismissed as something no longer an issue for a number of reasons. Firstly, in 2006 the International Agency for Research on Cancer (IARC) classified inorganic lead compounds as ***probably carcinogenic*** to humans (Group 2A) (IARC, 2006), meaning that any exposure to lead might be harmful. Secondly, there is increased evidence to suggest that lead causes a range of non-cancer related detrimental health effects at lower exposure levels. Recent systematic reviews and meta-analysis studies have provided a convincing amount of evidence to show that there is a greater concern for exposure to lower lead levels than previously thought. The US National Toxicology Program (NTP 2012) also stated that there is *sufficient* evidence that blood lead levels <10 μg/dl and <5 μg/dl are associated with adverse health effects in adults and children respectively (NTP National Toxicology Program, 2012). The adverse health effects include neurological, immunological, cardiovascular including hypertension, renal, and/or reproductive and developmental effects. These reasons mean that whilst levels are lower than they were, the risk from exposure is not completely eliminated and remains a concern.

The exposure to lead in the workplace may have reduced through better controlled processes or increased automation but it remains a fact that lead is still in demand globally. Eurobat, the association of European battery manufacturers, claims that lead-based batteries are the dominant technology in the global rechargeable market and that demand will keep growing in absolute terms. The main reason for this is that the lead used in lead-based batteries operates in a closed loop, with 99% of a battery’s lead being recoverable, which can then be re-used in new batteries (Eurobat, 2020). The International Lead Association (ILA) quote that lead ores are mined at a rate close to 5 million tonnes a year and the world market for refined lead is approximately $15 billion (International Lead Association, 2020). There is also secondary production, where lead is recovered from recycled products or from residues arising from the production process; this accounts for more than half of all lead processed throughout the world. In the US more than 80% of lead comes from secondary production with Europe reporting over 60% (International Lead Association, 2020).

### Mercury

The World Health Organisation (WHO) lists mercury as one of their top ten chemicals of concern (World Health Organisation, 2020). The United Nations Environment Programme (UNEP) approved a global mercury treaty which resulted in The Minamata Convention on Mercury in 2013 (United Nations Environment Programme, 2019), with the aim to reduce the use of mercury and mercury compounds before 2020 (United Nations Environment Programme, 2020). The main priorities of this were to phase out the existence and use of mercury mines, phase out or restrict mercury in products and industrial processes and to control the release of mercury into the environment (World Health Organisation, 2020). The background reference range of urinary mercury in GB has been reported as 1.4 μmol/mol creatinine (Morton et al., 2010) which is similar to levels published in other countries (Hoet et al., 2013; Centers for Disease Control and Prevention (CDC), 2012; Castaño et al., 2019). The ingestion of methyl mercury, predominantly from fish and other seafood, constitutes the main source of dietary mercury exposure in the general population (CDC, 2021).

The reduced use of mercury in a range of industrial applications is reflected in the occupational exposures shown in this dataset. As shown in Figure 3, in 2019, the P90 was only marginally above the background reference range, although some exceedances of the BMGV still occur.

Industries and companies such as dental practices, rubber and plastic manufacturing, lighting and neon sign manufacture/maintenance and the manufacture/maintenance of mercury-based measurement equipment have seen a decline in the number of samples sent for analysis. This is most likely due to the Minamata Convention. Companies who manufacture and maintain mercury-based measurement equipment have seen the biggest decline of both sample numbers and exposure levels. Until 2005, the average number of samples submitted from this sector was 113 per annum, whereas from 2014 onwards, the average has only been 17 samples per year. It is worth noting that the dataset is biased by a single, dominant company. A significant reduction in urinary mercury concentrations has been observed in this sector also; the 6 year period 1996–2001 the P90 was 24.4 μmol/mol creatinine, (above the GB guidance value), this was followed by 10.6 μmol/mol creatinine (2002–2007), 8.5 μmol/mol creatinine (2008–2013), and subsequently 2.5 μmol/mol creatinine (2016–2019). It is of note that in this sector there has not been a urinary mercury value above the GB guidance value since 2005.

In contrast others industries have seen growth, for example the waste and recycling sector. From 1996 to 1999 the laboratory received only six urinary mercury samples from the waste and recycling sector. In the next 10 years (2000–2009) the laboratory received 358 samples and the last 10 years (2010–2019) this has increased to 783 urinary mercury samples. This sector also continues to show a pattern of elevated exposures and not year on year reductions in exposure like most other sectors. In this sector, reviewing data within the 24 year period, in 17 of those years the P90 was above the background unexposed reference range. In addition the maximum concentration in four of the years exceeded the UK guidance value of 20 μmol/mol creatinine.

Traditionally dental workers have had the potential for exposure to mercury amalgam. From 1996 to 2015 urinary mercury samples were received from 24 dental practices. In 5 out of the 24 dental practices no occupational exposure to mercury was observed from the urinary mercury analysis. Although only 103 samples have been received from dental practices, many of them showed exposure to mercury with 62 samples being above the background reference range and two of those above the BMGV of 20 µmol Hg/mol creatinine. However, there have been no samples received from dental practices since 2015. Mercury levels were known to reduce when using pre-dosed encapsulated amalgam rather than reusable capsules and by 2009, 90% of Scottish dentists reported using encapsulated amalgam (Duncan et al., 2011). To reduce the release of mercury into the environment, from January 2019, dental amalgam must be in a pre-dosed encapsulated form and amalgam separators (device to capture and remove amalgam particles from wastewater) are now mandatory.

Although the laboratory has seen a decline in sample numbers from the mercury lighting sector, it has been suggested that this sector is undergoing market growth due to an emphasis on improving energy efficiency (Zimmermann et al., 2014; Gul et al., 2020). Fluorescent and other mercury containing bulbs and lamps are more energy efficient than incandescent and halogen lamps; this has resulted in a significant increase in use in recent years (Duncan et al., 2011; Zimmermann et al., 2014; Gul et al., 2020). This in turn has had an impact on the waste and recycling industry, leading to the creation of specific waste facilities capable of handling mercury waste with spent lamps/bulbs falling within the scope of the WEEE (waste electronic electrical equipment) directive (Zimmermann et al., 2014). Several studies have highlighted occupational exposure to mercury from the production and/or WEEE waste recycling of fluorescent lamps (Julander et al., 2014; Visnjevec et al., 2014; Zimmermann et al., 2014; Lecler et al., 2018; Gul et al., 2020) including a mercury poisoning case study of a fluorescent lamp glass blower in the North West of England (Guthrie et al., 2006) and subsequent observations of mercury exposure in a small group of glass blowers at a separate company where mean urinary mercury concentrations were 8.1 µmol Hg/mol creatinine (Guthrie et al., 2006).

Samples received at the laboratory from the waste and recycling sectors include those dealing with overall waste such as companies handling general domestic waste, commercial waste and recycling centres to more specific or specialist waste such as WEEE waste, clinical and chemical waste, waste oil and fuel, mercury lamps, fluorescent lamps/bulbs and liquid mercury waste. Samples specifically from WEEE and fluorescent lamp/bulbs recycling companies (n = 254, median 4.6 µmol Hg/mol creatinine, P90 30.3 µmol Hg/mol creatinine) show greater exposure to mercury than those from general waste and recycling companies (n = 621, median 0.6 µmol Hg/mol creatinine, P90 3.1 µmol Hg/mol creatinine).

### Benzene

Benzene exposures presented here, as assessed by urinary SPMA levels, have generally been steadily reducing over time. These reductions mirror general reductions in exposure across the wider environment (for example, the US EPA reported that the median ambient benzene concentrations halved (from 2 μg/m^3^) (US EPA, 2010) between 1994 and 2009) as well as improvements to workplace exposure control. A few studies have documented workplace benzene exposure over time, largely in the petrochemical sector (Capleton and Levy, 2005; Williams et al., 2005; Gaffney et al., 2010; Sahmel et al., 2013). In the time period spanning the last 50 years, these studies show that most of the benzene exposures were within the appropriate occupational exposure limit (OEL).; Moreover, many of the results were within contemporary OELs. Of course, these data sets may represent workplaces that have always demonstrated good exposure control and there are huge uncertainties involved in attempting to extrapolate individual data sets to the wider world. However, while improvements to work practices in traditionally high-exposure workplaces (such as oil and gas) appear to have led to declines in exposure, inadvertent exposures from operations such as engineering works on previously contaminated brownfield sites continue to be potentially significant exposure sources (Jones and McCallum, 2011). Isolated workplace incidents will inevitably remain a possible source of acute elevated exposure.

Excluding two instances (in 1997 and in 2008) of high-level exposures and intensive sampling campaigns from tunnelling through contaminated land (Jones and McCallum, 2011), our data can be grouped into five main categories. About a third of samples were from miscellaneous sources including chemical manufacturers, occupational health providers and sub-contracting laboratories. Another third came from the oil and gas industry, both on and offshore, and also from around the world. About 20% came from coke oven workers and 5% from foundries (using certain binders, which can release benzene when heated, in their casting moulds). These last samples came primarily from an HSE research study looking at chemical exposures in foundries (Cooke et al., 2017). A further 5% of samples came from the waste and recycling sector. Although the oil and gas sector made up a third of the samples overall, samples were only received in significant numbers (>100/year) since 2010; this may reflect the growing industry interest in benzene at this time with bodies such as Concawe and the Energy Institute funding research into benzene exposures and toxicology (Sams and Jones, 2015; Cox et al., 2017; Frigerio et al., 2020) The P90 in the oil and gas sector has remained consistent over the last 10 years (∼1.5 μmol/mol creatinine between 2009 and 2019); about 1.6% of results exceeded the ACGIH BEI (12 μmol/mol creatinine). For coke oven workers, most samples were received between 2007 and 2014, since then there has been a steady decline in samples due to loss of UK industry in this sector; during this time, the P90 fell from 6.8 to 2.6 μmol/mol creatinine. In total there have been only around 500 samples received from the foundry sector, mostly between 2009 and 2013 and coming from the HSE research project. During that time the P90 was fairly stable at 3.7–5.8 μmol/mol creatinine although a maximum result of 183 μmol/mol creatinine was detected. In the waste and recycling sector (296 samples), most were received since 2014 and the P90 was 2.7 μmol/mol creatinine.

Overall (excluding data from the two tunnelling incidents), the P90 has decreased from 15.9 (1997–2001) to 9.0 (2002–2006) to 3.6 (2007–2011) to 2.3 (2012–2016) to 1.8 μmol/mol creatinine in the last 3 years, demonstrating a continuous decline in exposures. Despite this decline, high exposures are still possible and about 4% of samples in the past decade have exceeded the ACGIH BEI, with some isolated extreme values. In the past 2 years, no results above 8 μmol/mol creatinine have been recorded. This is comparable with recent reported levels across a variety of workplaces including coke oven workers (Frigerio et al., 2020), gas station attendants (ECHA, 2018; Kraus et al., 2019) firefighters (Rosting and Olsen, 2020) and an oil spill field trial at sea (Gjesteland et al., 2018).

Having been a long-standing recognised carcinogen (IARC, 2018), there has been consistent pressure to minimise exposure to benzene through improvements to work practices and restrictions in its use. Since 2001, the ACGIH (ACGIH, 2001) has recommended a BEI for SPMA of 25 μg/g creatinine (12 μmol/mol creatinine) as a biomonitoring equivalent of exposure to 0.5 ppm benzene in air. In non-smokers, an air benzene level of 0.2 mg/m^3^ (0.06 ppm) corresponds to 3 µg SPMA/g creatinine (1.4 μmol/mol) (Schnatter et al., 2020). In 2018, the ECHA Committee for Risk Assessment published an opinion on benzene, recommending a BLV of 2 µg SPMA/g creatinine (0.9 μmol/mol creatinine, equivalent to 0.16 mg/m^3^ benzene in air) and in response to recent health concerns, the Committee for Risk Assessment (RAC) has recommended lowering the occupational exposure limit (OEL) to 0.05 ppm benzene in air based on chromosomal damage in bone marrow (Fustinoni et al., 2010). Meanwhile, following a comprehensive review of toxicology data, Schnatter *et al*, proposed an OEL of 0.25 ppm (8 h TWA) and derived NOAEL (0.5 ppm) and LOAEL (2 ppm) (Schnatter et al., 2020).

As better exposure control and regulatory pressure drive reductions in guidance values, it is becoming increasingly difficult to distinguish workplace exposure from other environmental sources of benzene using biological monitoring. Its presence in vehicle exhaust and tobacco smoke make benzene a ubiquitous environmental pollutant. While most environmental exposures remain well below current occupational exposure limits, SPMA levels in smokers can reach 3 μmol/mol creatinine (Fustinoni et al., 2010; Bevan et al., 2012). If the proposed reductions to OELs become reality, the use of biological monitoring for assessing occupational exposure to very low-levels of benzene is likely to become increasingly problematic. Due to the nature of products being handled at many workplaces where benzene exposure may occur, these sites are often non-smoking which might help to minimise the effect of recent exposure to tobacco smoke. The relative importance of the number of cigarettes smoked daily versus time since most recent cigarette is difficult to quantify. Some strategies to help distinguish workplace exposure to benzene might include establishing a non-work baseline, or asking individuals to record either the number of cigarettes smoked or the time of the last cigarette. However, at best, these approaches can only be semi-quantitative. Quantifying levels of the nicotine metabolite cotinine in urine samples has been proposed as a promising approach to separate out benzene exposure from tobacco smoke (Fustinoni et al., 2010; Lovreglio et al., 2018). The measurement of cotinine can be performed concurrently with SPMA analysis, thus minimising the additional laboratory effort required. However, it is important to consider the potential confounding effect of nicotine exposure from other sources, principally from nicotine-replacement devices such as patches, gum and vapes. Biological monitoring remains an important tool to help with the assessment and control of exposure, especially where control relies on the use of personal protective equipment, so it will be important to develop solutions to help to distinguish workplace exposure to benzene from other sources (mainly tobacco smoke).

The data presented here in Figure 4 and Table 1 for urinary SPMA reflects much of the literature and indicates that, generally, results are within current available guidance values. However, there are examples where improvement to exposure control is still required. Furthermore, benzene exposure can also occur beyond those workplaces that have traditionally been associated with petrochemicals or combustion products. Exposure to construction workers on previously contaminated sites or during remediation could become an increasing concern in the future.

### Hexamethylene Diisocyanate

HDI, like other di-isocyanates, is a potent asthmagen, with good exposure control needed to prevent sensitisation of workers. The use of biological monitoring is advantageous, for the motor vehicle repair sector in particular, due to the reliance on respiratory protective equipment to control workers’ exposure. Biological monitoring for HDI exposure (by quantifying the corresponding amine metabolite, HDA, in urine) became available in the mid-1990s (Maître et al., 1993; Williams et al., 1999). HSE has been actively involved in biological monitoring HDI exposure since then and there has been significant growth in demand for commercial analysis up to current levels of around 1,500 samples per year (Jones et al., 2013). Substantial regulatory intervention from HSE focussing on the motor vehicle repair sector is likely to have helped to establish the use of biological monitoring as a tool to help with exposure assessment. There is also good evidence from the data presented here, and as discussed by Jones *et al* (Jones et al., 2013), that these interventions successfully contributed towards reducing workplace exposure; P90 levels of 2.2 μmol/mol creatinine in 1995–2001 (prior to the national intervention study) were reduced to 0.7 μmol/mol creatinine in 2002–2013. However, levels have since increased again to 1.0 μmol/mol creatinine (2014–2019), which may reflect the greater number of workers sampled. There will also have been a turnover of workers since the national intervention (ended in 2008), so many current workers will not have received the messages of the intervention directly. The increased sample numbers may also, in part, reflect a broadening of workplaces using biological monitoring–many of which may also not have benefited from the national intervention messaging. Biological monitoring of isocyanate exposure remains a rather niche application, with only a few laboratories worldwide offering analysis. This is reflected in a low number of published studies (Scholten et al., 2020). An analysis of HSE data strongly suggested a link between reductions in HDI exposure and decreased reported cases of occupational asthma (Stocks et al., 2015), however recent biological monitoring data of US construction workers applying metal structure coatings demonstrated that 58% of samples exceeded 1 μmol/mol creatinine (Bello et al., 2020). These findings agree with our data and are suggestive that isocyanate exposures could still be further reduced in many cases.

Within the European Union, the Commission has proposed a REACH restriction on diisocyanates (ECHA Diisocyantes, 2021). This will mostly limit the concentration of diisocyanates in formulations to less than 0.1% unless workers receive specific training, regularly updated, in their safe use. The HSE intervention study was in fact cited in the restriction proposal as a demonstration of how awareness training can reduce exposures. ECHA is currently developing an opinion on occupational exposure limits for diisocyanates (ECHA, 2021); they are not proposing a limit but recommend that the approach to derive an exposure response for respiratory sensitisation be further developed. They also conclude that no BLV can be established due to “no reliable correlation between diisocyanate biomarkers and diisocyanate airborne levels, especially at low exposure levels”. It therefore seems that, in the short-term, using biological monitoring to monitor and reduce diisocyanate exposures in Europe will rely on national legislation and guidance (e.g. German BAT (Deutsche Forschungsgemeinschaft, 2020) of 15 μmol/mol creatinine).

## Conclusion

Most of the samples in the HSE database come without any contextual information; the samples are mainly commercial and the database used is primarily designed to store and process biological monitoring data only. This means there is limited scope to improve the collection and codified storage of such contextual information and this restricts how far the exposure data can be interpreted with respect to causal factors. Data may be biased downwards if the samples come only from ‘good’ workplaces that routinely monitor their employees or could be biased high by companies with exposure problems or in the case of blood lead where those with elevated levels are sampled more frequently under CLAW. Gradually reducing levels for lead, benzene and mercury may in part show the impact of national, regional and global regulatory action, respectively, although the observed general reduction in exposures through the potential decline of some industries as well as the increase in automation also need to be considered. The results for HDI show that whilst interventions can reduce exposures significantly, if the controls require worker training or behaviour change then the initiatives may need to be refreshed at intervals to maintain the reductions in exposure. We have observed that exposures move between sectors over time and that waste and recycling (lead, mercury) and remediation (benzene) sectors may be increasingly areas of concern as the numbers of workers and applications increase. Finally, it is apparent from the decreasing levels across the different substances discussed that the guidance values should be more frequently reviewed. In terms of mercury, the GB guidance value is a health based guidance value and as such this remains relevant but there is certainly a disparity in the blood lead and SPMA levels being measured in the most recent datasets and any relevant guidance values.

## Data Availability

The data analyzed in this study is subject to the following licenses/restrictions: Not available outside of organisation. Requests to access these datasets should be directed to email main author.

## Footnotes

1 RIQAS External Quality Assurance Programme https://www.randox.com/human-urine-eqa/
